# Panic disorder and incident coronary heart disease: a systematic review and meta-analysis protocol

**DOI:** 10.1186/s13643-015-0026-2

**Published:** 2015-03-25

**Authors:** Phillip J Tully, Gary A Wittert, Deborah A Turnbull, John F Beltrame, John D Horowitz, Suzanne Cosh, Harald Baumeister

**Affiliations:** Department of Rehabilitation Psychology and Psychotherapy, Institute of Psychology, University of Freiburg, Engelbergerstr. 41, Freiburg, 79085 Germany; Freemasons Foundation Centre for Men’s Health, Discipline of Medicine, School of Medicine, The University of Adelaide, 254 North Terrace, Adelaide, Australia; Department of Cardiology, Basil Hetzel Institute, Queen Elizabeth Hospital, University of Adelaide, 28 Woodville Road, Adelaide, Australia; Clinic of Psychiatry and Psychotherapy II, University of Ulm, Helmholtzstr, Gunzburg, Germany

**Keywords:** Panic disorder, Panic attack, Anxiety disorder, Anxiety neurosis, Coronary heart disease, Myocardial infarction, Systematic review, Meta-analysis, Protocol, Etiology

## Abstract

**Background:**

The clinical presentation of panic disorder and panic attack overlaps many symptoms typically experienced in coronary heart disease (CHD). Etiological links between panic disorder and CHD are controversial and remain largely tenuous. This systematic review aims to pool together data regarding panic disorder with respect to incident CHD or myocardial infarction.

**Methods/Design:**

Electronic databases (MEDLINE, EMBASE, PsycINFO and SCOPUS) will be searched using a search strategy exploding the topics for CHD and panic disorder. Authors and reference lists of included studies will also be contacted to identify additional published and unpublished studies. Eligibility criteria are as follows: Population: persons without CHD who meet criteria for panic disorder, panic attack, anxiety neurosis or elevated panic disorder symptoms; Comparison: persons without CHD who do not meet criteria for panic disorder, panic attack, anxiety neurosis or elevated panic disorder symptoms; Outcome: verified fatal and non-fatal CHD at follow-up; including coronary revascularization procedure, coronary artery disease, and myocardial infarction. Studies adopting self-report CHD will be ineligible. Screening will be undertaken by two independent reviewers with disagreements resolved through discussion. Data extraction will include original data specified as hazard ratios, risk ratios, and original cell data if available. Risk of bias assessment will be undertaken by two independent reviewers. Meta-analytic methods will be used to synthesize the data collected relating to the CHD outcomes with Cochrane Review Manager 5.3.

**Discussion:**

This systematic review aims to clarify whether panic disorder is associated with elevated risk for subsequent CHD. An evaluation of the etiological links between panic disorder with incident CHD might inform evidence-based clinical practice and policy concerning triaging chest pain patients, diagnostic assessment, and psychiatric intervention with panic disorder patients.

**Systematic review registration:**

PROSPERO CRD42014014891.

**Electronic supplementary material:**

The online version of this article (doi:10.1186/s13643-015-0026-2) contains supplementary material, which is available to authorized users.

## Background

Panic disorder is among the most prevalent mental health conditions in the community [1] and primary healthcare practice [2], collectively signifying a major public health burden in terms of economic costs [3,4], quality-adjusted life years, and disability worldwide [5]. Panic disorder is characterized by recurrent and unexpected panic attacks; that is, an abrupt surge of intense fear or discomfort reaching a peak within minutes, during which at least four of the symptoms listed in Table 1 are experienced in conjunction with persistent concern or worry about additional panic attacks or their consequences, or maladaptive behaviors including avoidance [6]. Strictly speaking, psychiatric diagnostic nomenclature indicates that a panic disorder diagnosis cannot be applied when panic symptoms are the direct result of a medical condition such as CHD [6]. Yet many symptoms characteristic of a panic attack overlap with the clinical presentation of coronary heart disease (CHD) [7] and cardiomyopathies [8], making differential diagnosis difficult [9]. For example, chest pain and dyspnea are panic-like symptoms yet also overlap with those typical of a myocardial infarction (MI) and angina pectoris. Given the commonality in clinical presentation of panic attacks and CHD, it has been speculated that panic disorder is linked with CHD for more than 50 years [10]. However, the nexus between panic disorder and CHD remains tenuous [11,12] and is yet to be clarified by means of a contemporary systematic review.

**Table 1:** **List of possible symptoms experienced during a panic attack**

**Number**	**Panic disorder symptom**
1	Palpitations, pounding heart, or accelerated heart rate
2	Sweating
3	Trembling or shaking
4	Sensations of shortness of breath or smothering
5	Feelings of choking
6	Chest pain or discomfort
7	Nausea or abdominal distress
8	Feeling dizzy, unsteady, light-headed, or faint
9	Chills or heat sensations
10	Paresthesias (numbness or tingling sensations)
11	Derealization (feelings of unreality) or depersonalization (being detached from oneself)
12	Fear of losing control or ‘going crazy’
13	Fear of dying

Panic disorder/panic attack symptoms adapted from the Diagnostic and Statistical Manual of Mental Disorders Fifth Edition [6]*.*

Additional qualifiers:

A. A panic attack is an abrupt surge of intense fear or intense discomfort that reaches a peak within minutes, and during which time four (or more) of the above symptoms occur.

B. At least one of the attacks has been followed by 1 month (or more) of one or both of the following:

1. Persistent concern or worry about additional panic attacks or their consequences (for example, losing control, having a heart attack, ‘going crazy’).

2. A significant maladaptive change in behavior related to the attacks (for example, behaviors designed to avoid having panic attacks, such as avoidance of exercise or unfamiliar situations).

A. The disturbance is not attributable to the physiological effects of a substance (for example, a drug of abuse, a medication) or another medical condition (for example, hyperthyroidism, cardiopulmonary disorders).

B. The disturbance is not better explained by another mental disorder (for example, the panic attacks do not occur only in response to feared social situations, as in social anxiety disorder; in response to circumscribed phobic objects or situations, as in specific phobia; in response to obsessions, as in obsessive-compulsive disorder; in response to reminders of traumatic events, as in posttraumatic stress disorder; or in response to separation from attachment figures, as in separation anxiety disorder).

Part of the suspicion concerning such etiological links is that persons with panic disorder persist in emergency department recidivism [13,14] and outpatient examinations for chest pain [15,16] despite negative diagnostic results from coronary catheterization, electrocardiogram, or serum markers of myocardial damage [17,18]. These collective findings contrast to more recent evidence revealing plausible mechanisms of cardiopathogenesis attributable to panic disorder and sympathetic discharge of panic attacks, including reversible myocardial ischemia [19,20], diminished heart rate variability (HRV) [21], change in the QRS complex [22], especially the QT-interval [23-25], serum low density lipoprotein [26], microvascular disorders including coronary slow-flow [27] and microvascular angina [15], arterial stiffness [28], and also a preponderance of behavioral factors such as smoking [29], alcohol use [30], and overt exercise-avoidance behaviors [31]. The inconsistency in collective findings to date, coupled with the high coronary healthcare utilization by panic disorder patients [32], indicates that a meta-analysis is both timely and warranted.

Prior reviews concerning panic disorder and CHD have typically been narrative [11,33-35] or have performed meta-analysis on panic disorder prevalence in CHD [9,12,36] but none have quantified possible etiological links. Most recently, a meta-analysis of 20 studies reported by Roest and colleagues [37] suggested that anxiety was associated with a 26% increased risk of CHD and a 48% increased risk of CHD mortality. However, this previous review [37] is confounded by numerous methodological shortcomings, thereby limiting the extent to which conclusions regarding anxiety disorders and incident CHD can be drawn. Firstly, the meta-analysis included cohorts comprised by persons with CHD at baseline (six studies), mixing etiological and prognostic studies, thereby precluding an evaluation of etiological links. A second limitation is the inclusion of studies utilizing self-report to ascertain CHD outcomes [38], which is notably problematic in anxiety disorder patients because of their propensity towards catastrophic misinterpretation of somatic symptoms [39], and the aforementioned high utilization of emergency departments [32], thus underscoring the requisite need to prioritize physician diagnoses when establishing etiological links [39].

Notwithstanding these methodological dilemmas, further limitations of the extant evidence base relate to key conceptual and measurement issues. Specifically, the prior etiological review concerning anxiety [37] included only one study adopting anxiety disorder diagnoses (that is, generalized anxiety disorder), no studies concerning panic disorder, and excluded several studies pertaining to anxiety disorders among non-institutionalized individuals [40,41]. Therefore, collectively, the prior findings [37] do not represent the anxiety disorder subtypes observed in clinical practice, and especially those necessitating psychiatric intervention [42]. Rather, the prior meta-analysis [37] has pooled together broad anxiety domains measured by self-report anxiety questionnaires. One drawback of explicating etiological links in this manner is that questionnaires measure quite discrepant anxiety phenotypes, as for example uncontrollable worry is characteristic of generalized anxiety disorder, intrusive obsessions are characteristic of obsessive-compulsive disorder. By contrast, other self-report measures purported to measure anxiety do not characterize anxiety *per se* but rather tend to measure personality traits (see [43-45]) or non-specific negative emotions shared with depression (see [46-48]). For example, the Hospital Anxiety and Depression Scale (HADS), used in two cohorts, is widely documented for the undesired psychometric inability to distinguish anxiety from depression, raising serious concerns about construct validity [49]. In light of the epidemiological and clinical implications, it is therefore requisite to examine anxiety at the specific disorder and phenotype level rather than as a single heterogeneous group, thus allowing for the considerable taxonomic, phenotypic, and genetic heterogeneity to be accounted for [9].

A final limitation of the extant evidence basis concerning anxiety is that the conjoint effects of anxiety and depression with respect to incident CHD is unknown. Concurrent and lifetime comorbidity between anxiety and depression is common [50], and some studies have reported that comorbid anxiety and depression disorder is associated with a higher CHD risk than either disorder in isolation [51]; however, such studies have not been subjected to systematic review and meta-analysis. Collectively, the inconsistencies in prior findings highlight a critical limitation with respect to clinical diagnosis of panic disorder and etiology with CHD. A systematic review of this type, as described herein in a protocol stage, might in turn assist in the design of subsequent epidemiological studies and inform clinicians. Herein, we outline a systematic review and meta-analysis protocol designed to overcome the abovementioned limitations pertaining to panic disorder and CHD.

## Methods/Design

### Aims

The proposed review aims to synthesize the evidence base regarding panic disorder and subsequent CHD. The reporting of this review will conform to the PRISMA guidelines [52].

### Search strategy

We will identify relevant articles in any language by searching electronic databases from inception including: MEDLINE, EMBASE, SCOPUS, and PsycINFO. The search strategy is provided in Additional file 1. We will perform a hand search of the reference lists of articles selected to supplement the electronic search. In addition, a hand search will be performed of prior narrative reviews concerning panic disorder [11,12,53]. The principal investigators of studies will also be contacted to ascertain unpublished data and determine other studies not yielded by our primary search. The grey literature/unpublished studies will not be searched on an electronic database.

### Eligibility criteria

Population: The population of interest are persons with panic disorder at baseline but without verified or known CHD at this time. To be eligible, panic disorder must be reported according to a recognized clinical criteria, including the Diagnostic and Statistical Manual of Mental Disorders (DSM) or the International Classification of Diseases, or determined by a standardized interview (for example, Structured Clinical Interview, Composite International Diagnostic Interview), or diagnosis made by a qualified professional (for example, psychiatrist, psychologist, general physician), or medical records. Studies reporting panic disorder ± agoraphobia/phobic neurosis, panic disorder ± other anxiety disorder, or a validated self-report measure of panic attack or panic attack symptoms, or anxiety neurosis, the diagnostic precursor to panic disorder in ICD, are eligible. In the case that studies adopt a measure of general anxiety, we specify that at least 85% of items have to be deemed by blind reviewers as symptomatic of PD to be included in the analyses. Single-item measures are eligible only if they explicitly refer to a panic attack or panicky sensation. Studies are eligible if reporting from the general, cardiology, or psychiatric population (in- and outpatients). Studies must be performed among young adults (≥15 years) or adult populations (no upper limit of age for inclusion).

Comparator/control: Participants without verified or known CHD, and without panic disorder, at baseline from the general, cardiology, or psychiatric population (in- and outpatients).

Outcomes: Incident CHD will be considered as follows:

1. Major adverse cardiac events - defined as documented death due to CHD, cardiac arrest (including ventricular fibrillation), sudden cardiac death or myocardial infarction (fatal or non-fatal).
2. Structural coronary artery disease - as evidenced by obstructive coronary artery disease (≥50% stenosis) on coronary angiography and/or subsequent coronary revascularization.
3. Ischemic heart disease - clinical evidence of myocardial ischemia on ECG (transient ST/T wave changes), myocardial scintigraphy (reversible defect), echocardiography (transient wall motion abnormality), or cardiac magnetic resonance imaging (perfusion defect or transient wall motion abnormality), either during a spontaneous episode or a provocative stress stimulus.
4. Other CHD - physician or cardiologist diagnosed CHD.

We will stratify the primary CHD endpoint as any CHD (CHD endpoint levels 1 to 4), fatal CHD (verified death for CHD, levels 1 to 4), and fatal or non-fatal MACE (CHD level 1).

Study design: Only peer reviewed studies in full-text, conference abstract, or doctoral dissertations are eligible for this review if published in English. Observational studies designed as longitudinal cohort, case–control study, or database registry and experimental studies designed as randomized controlled trials or non-randomized trials will be eligible for this review. Prospective and retrospective studies will be eligible. We will exclude cross-sectional studies, case series, and case reports.

Exclusion: Studies utilizing only patient self-report to determine incident CHD, including self-report of a physician diagnosis. Studies reporting neurocirculatory asthenia, cardiac neurosis, effort syndrome, or Da Costa’s syndrome are ineligible as they precede panic disorder [10] or are re-conceptualized under multiple psychosomatic disorders [54].

### Study selection process

Initially, two reviewers (PJT, SC) will independently screen titles and abstracts of all the retrieved bibliographic records. Full texts of all potentially eligible records passing the title and abstract screening level will be retrieved and examined independently by the two reviewers according to the abovementioned eligibility criteria. Disagreements at both screening levels (title/abstract and full text) will be adjudicated by discussion with a third reviewer (HB). A PRISMA flow chart will outline the study selection process and reasons for exclusions.

### Data items for collection

After determination of the initial study, eligibility information will be extracted for each study pertaining to study identification (first author, year of publication, country where recruitment took place), study design characteristics (sample size, duration of follow-up), patient population (age, gender, proportion with comorbid hypertension, hypercholesterolemia and diabetes), the type of CHD endpoint (type of CHD, criteria for CHD), and adjustment for covariates (complete list of variables). Primary outcome data collected will include CHD, reported either as categorical numbers (numerator and denominator) or the statistical effect size (that is, risk ratio, hazard ratio, incidence rate ratio, odds ratio) and the 95% confidence interval (CI). These variables will be extracted for all studies by one reviewer (PJT), after which the extracted data will be verified by a second reviewer (SC) to reduce reviewer errors and bias. All disagreements will be handled by consensus between the three reviewers (PJT, SC, HB).

### Risk of bias

The RTI item bank will be utilized to identify methodological bias among the identified studies [55]. The RTI item bank consists of 29 items for evaluating the risk of bias in observational studies, interventions, or exposures. The RTI was developed from an initial pool of 1,492 items based on face validity, cognitive, content validity, and inter-rater reliability testing.

The scale has demonstrated inter-rater reliability. Risk of bias will be independently undertaken by two reviewers (PJT, SC) and disagreements adjudicated by consensus with a third reviewer (HB). The RTI item bank is provided in Additional file 2.

### Synthesis of data and summary measures

#### Data synthesis

We will provide a detailed description of the results in both tables and text for all included studies. We will qualitatively describe the studies pertaining to study identification (first author, year of publication, country where recruitment took place), study design and characteristics (observational or experimental, sample size, duration of follow-up), patient population (age, gender), the classification of panic disorder or symptoms (description of clinical interview or panic questionnaire), the type of CHD endpoint (type of CHD, criteria for CHD), the length of follow-up, and adjustment for covariates (list of variables).

### Meta-analysis

We will use RevMan 5.3 to conduct the meta-analyses. The summary effect measures may include hazard ratios, relative risk, or odds ratios. When data are available to be pooled together, we will use a random-effects model using the inverse variance method which provides a more conservative estimate of effect size. Where possible we will aggregate each included study’s CHD outcome data as hazard ratios, relative risk, or odds ratios with the associated 95% CIs as these are presumed to measure the same underlying effect [56] and consensus that these are approximately equivalent for effect sizes less than 2.5 and follow-up less than 20 years [57]. In studies where an effect size is not reported, we will extract the individual cell data and calculate the RR and 95% CI. In the first instance, we will pool together the unadjusted effect sizes for each CHD outcome (permitting age and sex adjustment). In the second instance, we will pool together the unadjusted and most adjusted effect sizes for each CHD outcome. Heterogeneity will be evaluated with the *I*^2^ statistic. According to the Cochrane Handbook for Systematic Reviews [58], *I*^2^ of 0% to 60% can be regarded as not important to moderate, while *I*^2^ > 60% indicates substantial heterogeneity.

### Handling of multiple endpoints

There is a likelihood of more than one reported eligible endpoint per study [59], we will include data as follows: 1. the CHD endpoint with the largest *N* will be prioritized, then fatal and non-fatal MACE, then obstructive coronary artery disease, ischemic heart disease, and clinician diagnosed CHD; 2. in the case where several CHD outcome measures of the same hierarchy level are used in one study, we will select the outcome measure that is used most frequently across the eligible studies, if possible.

### Planned subgroup analyses

Subgroup analyses will be performed for the primary CHD endpoint on psychiatric classification level (1) studies utilizing anxiety neurosis diagnoses *versus* all other anxiety classifications; (2) studies utilizing self-report symptoms *versus* all others; (3) studies utilizing panic disorder *versus* all others; (4) psychiatric inpatients [60] *versus* outpatients or community samples; (5) persons with panic comorbid with a depression disorder *versus* persons with panic only; (6) effect sizes adjusted for depression *versus* studies not adjusted for depression [61]; and (7) known psychosocial correlates and CHD risk factors [62] including effect sizes adjusted for alcohol; (8) adjusted for tobacco smoking; (9) adjusted for socioeconomic status.

We will also perform subgroup analysis stratified by groups of patients who undergo coronary catheterization with negative results at baseline *versus* all other groups of patients with indeterminate baseline CHD. We will also perform subgroup analyses stratified by gender if possible. In the event that stratified gender analyses is not possible, we will utilize meta-regression to perform analyses adjusted for the percentage of males/females in the total sample based on the preponderance to PD in females and preponderance to CHD in males, and age (mean or median). Sensitivity analyses will evaluate the effects of age <50 and >50 years, or alternatively as mean or median age in meta-regression. This is based on the *a priori* higher probability for older persons to have CHD, CAD, myocardial infarction, cardiomyopathy, and atrial fibrillation.

### Planned sensitivity analyses

Because of possible under- or overestimation of effect sizes, we will adopt Loef and Walach’s [56] methodology for sensitivity analyses and apply the natural logarithms of calculated values and calculate the standard errors based on 95% CIs.

Sensitivity analyses will be performed for the primary CHD endpoint, and we will assess general study-level characteristics as potential sources of heterogeneity (1) studies adjusting for reverse causation bias by excluding CHD events occurring in the first 2 years of follow-up; (2) retrospective studies; (3) region or country of recruitment; (4) unpublished studies; (5) length of follow-up. Due to the nature of CHD outcomes assessed in the review, it is possible that studies will report multiple observations with heterogeneity concerning the length of follow-up. We will analyze follow-up duration using different time frames: 1. short term (up to 2 years); 2. medium term (2 to 10 years); and 3. long term (more than 10 years). Our inclusion criteria do not specify requisite time points at which outcomes are measured to avoid excluding potentially useful information; however, we will account for timing during data analysis. We intend to group all studies together initially and then perform sensitivity analyses for different time points (for example, CHD in the short, medium, and long terms), if possible.

### Assessment of publication bias

The test of Egger *et al*. [63] and the funnel plot will be used to evaluate the presence of publication bias.

### GRADE framework for quality of evidence

The proposed review will use the Grading of Recommendations Assessment, Development and Evaluation (GRADE) guidelines [64] to determine the quality of evidence and the strength of recommendations. The GRADE guidelines will be applied separately to each of the CHD endpoints, providing a summary of findings table with qualitative description as either high, moderate, low, or very low.

## Discussion

This systematic review aims to add to the extant literature by aggregating data concerning the risk of incident CHD attributable to panic disorder in persons originally free from CHD. Our review will extend beyond previous systematic reviews including those concerning post-traumatic stress disorder [65] and, indeed, a prior review concerning anxiety symptoms [37]. Furthermore, the findings will potentially need to be considered alongside a plethora of psychosocial risk factors also purported to increase CHD risk, and these include depression [66-68], stress [69], anger and hostility [70], and Type A personality [71] to ensure uniqueness of the findings from related negative emotions [72]. The findings might therefore serve to clarify the design of future epidemiological and clinical studies and also potentially inform evidence-based clinical practice and policy concerning triaging chest pain patients [17], diagnostic assessment [8], and psychiatric intervention with panic disorder patients [73].

There are several limitations that will contextualize the findings and generalizability of the proposed review including the interrelatedness of psychiatric disorders especially anxiety and depression disorders [74,75]. Moreover, studies will potentially determine panic disorder status by different means such as clinical interview, physician consultation, or self-reported panic attack questionnaires. Therefore, the proposed review may be limited by the pooling together of panic disorder studies with varying levels of validity and heterogeneity. A related limitation concerns the CHD endpoints, and it is possible that different studies will utilize varying methods to determine CHD status at follow-up, each with varying levels of validity and conclusiveness. This limitation is important in our proposed review’s context since that we are largely assessing etiological links assuming that panic precedes CHD, when it is plausible that panic is merely a manifestation of undiagnosed cardiovascular dysfunction, coronary spasm, or sub-clinical CHD. Indeed, panic disorder diagnoses are sometimes applied mutually exclusively to CHD once exploratory diagnostic tests for myocardial infarction are negative [17]. Limitations of the original studies may also include between study heterogeneity and high risk of bias that will potentially limit the conclusions drawn. Specifically, it is possible that retrospective studies will be characterized by a high risk of bias. Finally, despite attempts to retrieve unpublished and nonsignificant studies, the proposed systematic review is likely to be limited by publication bias of only significant findings, given the infancy of the literature [76]. Moreover, as the proposed review will include only English language studies, the generalizability of the findings to studies published in other languages and other healthcare settings is limited.

In conclusion, given that panic disorder and incident CHD links are tenuous and the absence of a contemporary meta-analysis on this topic, the proposed review will help in summarizing the available evidence. The findings may have implications for clinical practice and policy.

## Additional files

Additional file 1:
**Table showing the search strings for MEDLINE, EMBASE, PsychINFO and SCOPUS.** This table shows the search string for the systematic review for each of the databases utilized in our review, MEDLINE, EMBASE, PsychINFO, and SCOPUS. Additional file 2:
**RTI Risk of bias item bank.** This table shows each of the items of the RTI item bank used in our study. Each of the studies selected for full text review will be scored to these items by two reviewers.

## Abbreviations

CHD: coronary heart disease
CI: confidence interval
HR: hazard ratio
OR: odds ratio
RR: risk ratio
