# Supplementary material for: Panic disorder and incident coronary heart disease: a systematic review and meta-analysis protocol
Source: Syst Rev. 2015 Mar 25;4:33. doi: 10.1186/s13643-015-0026-2 (PMC4376084; doi:10.1186/s13643-015-0026-2)
Supplement: Additional file 1: — Table showing the search strings for MEDLINE, EMBASE, PsychINFO and SCOPUS. This table shows the search string for the systematic review for each of the databases utilized in our review, MEDLINE, EMBASE, PsychINFO, and SCOPUS. [file 13643_2015_26_MOESM1_ESM.pdf]

Additional File 1. Table showing the search strings for MEDLINE, EMBASE, PsychINFO and SCOPUS

MEDLINE	EMBASE	PsycINFO	SCOPUS
((((myocardial infarction[TIAB] OR coronary artery disease[MeSH Terms] OR coronary heart disease[MeSH Terms] OR ischemic heart disease[tiab] OR ischaemic heart disease[tiab] OR percutaneous coronary intervention[tiab] OR coronary artery bypass graft[tiab] OR CABG[tiab] OR CHD[tiab] OR cardiac death[tiab]))) AND (anxiety disorders[MeSH Terms] OR agoraphobia[MeSH Terms] OR phobic disorders[MeSH Terms] OR panic disorder[MeSH Terms] OR anxiety disorder[tiab] OR agoraphobia[tiab] OR phobic disorder[tiab] OR panic disorder[tiab] OR panic attack[tw]	('myocardial infarction'/exp OR 'infarction'/de) OR ‘coronary 'artery disease'/exp OR ‘coronary heart'/exp OR 'heart disease'/exp OR 'disease'/de OR ‘ischemic heart disease'/exp OR ‘ischemic heart disease'/exp OR percutaneous coronary intervention OR coronary artery bypass graft OR cabg OR chd OR ‘cardiac death'/exp AND anxiety NEXT/1 disorder* OR 'anxiety neurosis'/syn OR 'panic disorder'/de OR 'phobia'/de OR 'anxiety disorder'/de OR 'panic attack'/de OR 'agoraphobia'/de OR 'phobic' OR 'neurosis'/de	exp heart disorders/ or ischemic heart disease.mp. or coronary artery disease or myocardial infarct$ or heart infarct$ or coronary artery bypass or coronary heart disease or chd or CAD or coronary angioplasty or cardiac death<br><br>exp anxiety disorder$/ or anxiety neurosis.mp. or anxiety disorder.mp. or agoraphobia.mp. or anxiety neurosis.tw. or phobic neurosis.tw. or panic attack$.tw. [mp=title, abstract, heading word, table of contents, key concepts, original title, tests & measures]	(TITLE-ABS-KEY ("coronary artery disease") OR TITLE-ABS-KEY ("myocardial infarction") OR TITLE-ABS-KEY ("coronary heart disease") OR TITLE-ABS-KEY ("coronary angioplasty") OR TITLE-ABS-KEY ("coronary artery bypass") OR TITLE-ABS-KEY ("cardiac death") OR TITLE-ABS-KEY ("ischemic heart disease") OR TITLE-ABS-KEY ("ischaemic heart disease") AND TITLE-ABS-KEY ("anxiety disorder") OR TITLE-ABS-KEY ("panic disorder") OR TITLE-ABS-KEY ("panic attack") OR TITLE-ABS-KEY ("anxiety

OR anxiety neurosis [tw] OR phobic neurosis[tw])			neurosis") OR TITLE-ABS-KEY ("phobic neurosis") OR TITLE-ABS-KEY ("agoraphobia"))
